# Investigating crosstalk between H3K27 acetylation and H3K4 trimethylation in CRISPR/dCas-based epigenome editing and gene activation

**DOI:** 10.1038/s41598-021-95398-5

**Published:** 2021-08-05

**Authors:** Weiye Zhao, Ying Xu, Yufan Wang, Dan Gao, Jasmine King, Yajie Xu, Fu-Sen Liang

**Affiliations:** Department of Chemistry, Case Western Reserve University, 2080 Adelbert Road, Cleveland, OH 44106 USA

**Keywords:** Chemical modification, Epigenomics

## Abstract

Epigenome editing methods enable the precise manipulation of epigenetic modifications, such as histone posttranscriptional modifications (PTMs), for uncovering their biological functions. While histone PTMs have been correlated with certain gene expression status, the causalities remain elusive. Histone H3 Lysine 27 acetylation (H3K27ac) and histone H3 Lysine 4 trimethylation (H3K4me3) are both associated with active genes, and located at active promoters and enhancers or around transcriptional start sites (TSSs). Although crosstalk between histone lysine acetylation and H3K4me3 has been reported, relationships between specific epigenetic marks during transcriptional activation remain largely unclear. Here, using clustered regularly interspaced short palindromic repeats (CRISPR)/dCas-based epigenome editing methods, we discovered that the ectopic introduction of H3K27ac in the promoter region lead to H3K4me3 enrichment around TSS and transcriptional activation, while H3K4me3 installation at the promoter cannot induce H3K27ac increase and failed to activate gene expression. Blocking the reading of H3K27ac by BRD proteins using inhibitor JQ1 abolished H3K27ac-induced H3K4me3 installation and downstream gene activation. Furthermore, we uncovered that BRD2, not BRD4, mediated H3K4me3 installation and gene activation upon H3K27ac writing. Our studies revealed the relationships between H3K27ac and H3K4me3 in gene activation process and demonstrated the application of CRISPR/dCas-based epigenome editing methods in elucidating the crosstalk between epigenetic mechanisms.

## Introduction

The covalent histone post-translational modification (PTM) is one of the major epigenetic mechanisms where a variety of chemical functional groups can be dynamically added to or removed from both tails and globular domains of histones^1,2^. Genome-wide profiling and gene expression analysis enabled by the next generation sequencing (NGS) technologies have established the correlation between the distribution of certain histone PTMs with particular transcriptional status^3–6^. For instance, Histone H3 Lysine 27 acetylation (H3K27ac) preferentially resides at promoters and enhancers and is strongly correlated with active transcription^7–10^. Histone H3 Lysine 4 trimethylation (H3K4me3) is typically located around the transcriptional start site (TSS) of active genes, whereas active enhancers are not enriched with H3K4me3 but with histone H3 Lysine 4 monomethylation (H3K4me1)^9–12^. However, correlations between histone PTMs and gene activities mapped by these approaches cannot provide mechanistic information in these epigenetic regulatory processes and neither the causal relationships between histone PTMs and transcriptional activities^13,14^. Furthermore, combinations of histone PTMs have been proposed to constitute “histone codes” that cooperatively regulate gene expression^15–19^. The co-localization of H3K27ac and H3K4me3 at active promoters of active genes revealed by NGS analyses^7,12,20,21^ suggests potential interplay between these two histone PTMs in transcriptional regulation^18,19^. However, the distinct roles of these two active histone marks in transcriptional activation and the underlying molecular mechanisms of their interplay are still not fully understood^18^.

Several studies have revealed the crosstalk between H3K4me3 and histone acetylation. H3K4me3 was shown to facilitate H4K16ac enrichment in certain genomic contexts from loss-of-function studies of WDR5, a subunit of COMPASS H3K4me3 methyltransferase complex^22^. WDR5, however, is also a subunit in H3/H4 acetyltransferase complexes^23^, making it difficult to elucidate the causal relations of these two PTMs using this method^18^. Other studies carried out in *Drosophila* and human cells employing genetic manipulation approaches have revealed that H3K27ac and H3K4me3 are interdependent on each other^9,20^. However, how the roles and relationships between these two active marks during the process of transcriptional activation remain to be uncovered. Though pharmacological inhibition^24–26^ and genetic manipulation have facilitated the understanding of histone PTMs in epigenetic regulation, these methods cause global alteration of epigenome, resulting in complex secondary effects which may pose obstacles to elucidate the underlying epigenetic mechanisms.

Clustered regularly interspaced short palindromic repeats (CRIPSR)/dCas9-based epigenome editing technologies allow programmable locus-specific manipulation of epigenetic environment without global disruption, which is advantageous in investigating epigenetic regulation in gene activities^27–32^. Using this approach, p300 acetyltransferase have been targeted locus specifically to install H3K27ac at promoters and enhancers leading to transcriptional activation of targeted genes^33^. Furthermore, through a chemical inducible CRISPR/dCas9-based epigenome editing method, which allowed precise temporal mapping of epigenetic and gene expression events, the casual relationship between H3K27ac and transcription has been revealed^34^. It is known that crosstalk between different epigenetic marks and mechanisms collectively regulate gene activities^35,36^. Applying CRISPR/dCas9 and zinc finger protein-based methods, artificially installed H3K4me3 at permissive genome loci has been shown to enable sustained gene reactivation through the crosstalk between H3K4me3 and histone H3 Lysine 79 monomethylation (H3K79me)^35^.

**Figure 1:**
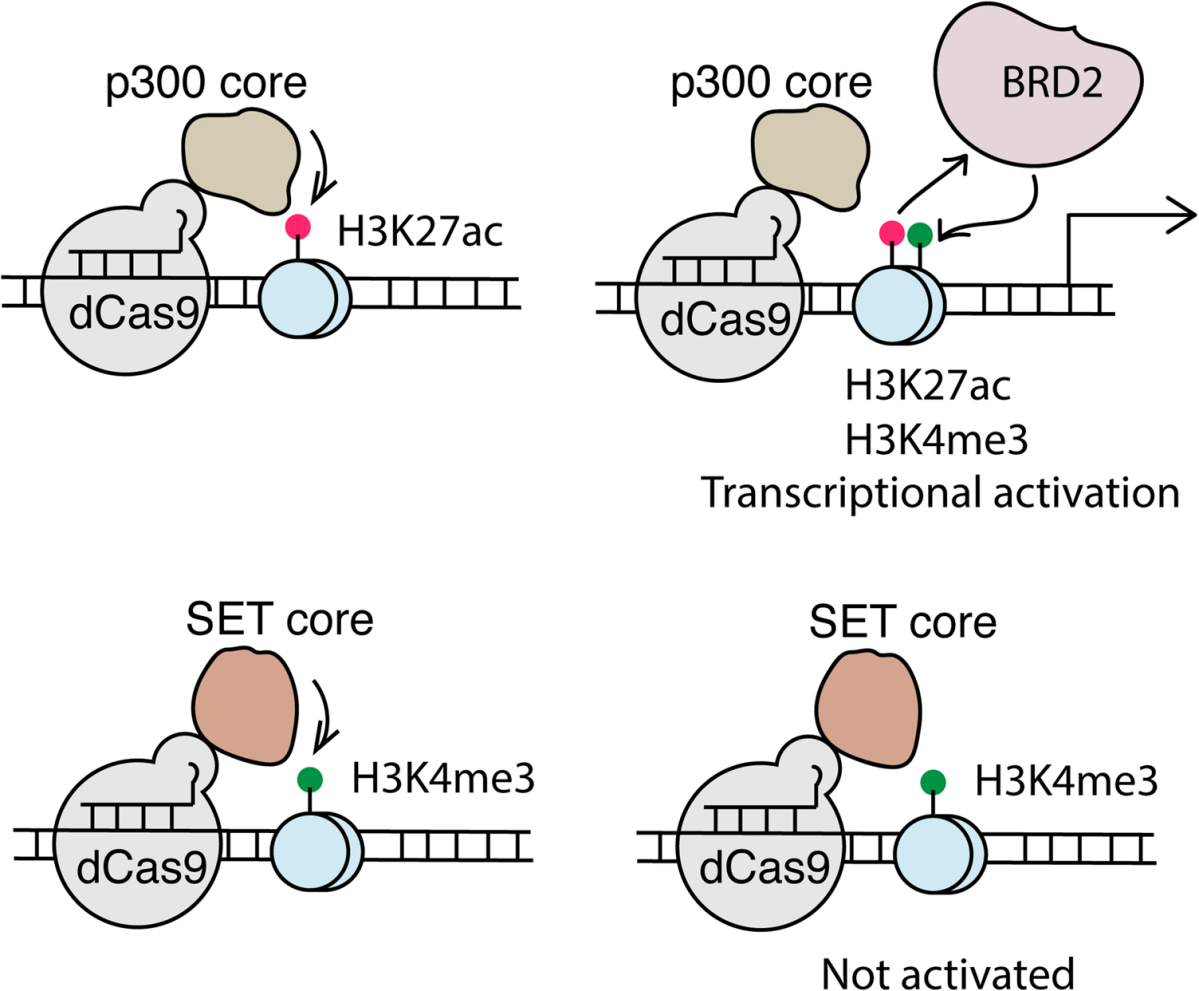
Investigating the H3K27ac/H3K4me3 crosstalk and functions through CRISPR/dCas9 based epigenome editing approaches.

Here we studied the crosstalk between H3K27ac and H3K4me3 around TSSs and their relationships and roles in transcription activation using the dCas-p300 fusion protein^33^ and a newly engineered protein dCas9-SET(CD), containing the core domain of SET1 (an H3K4me3-specific histone methyltrasferase^37–39^) (Fig. 1). Combining CRISPR/dCas9-based editing methods with pharmacological inhibition, we gained insights into the causal relationships between these two histone PTMs in gene activation and the associated molecular mechanisms. Our results suggest that H3K27ac is an upstream epigenetic mark which promotes H3K4me3 upregulation, but not the other way around, through acetyl-histone reader BRD2 and triggers transcriptional activation. Although H3K4me3 at promoters has been positively correlated with active genes^10^ and shown to reactivate gene expression^35^, we found that H3K4me3 is not sufficient for gene activation at the gene loci targeted by CRISPR/dCas9-based editing methods in this study. Collectively, we uncovered the crosstalk between H3K27ac and H3K4me3 and their roles in transcriptional activation.

## Results

### H3K27ac writing induced H3K4me3 enrichment and activated gene expression

**Figure 2:**
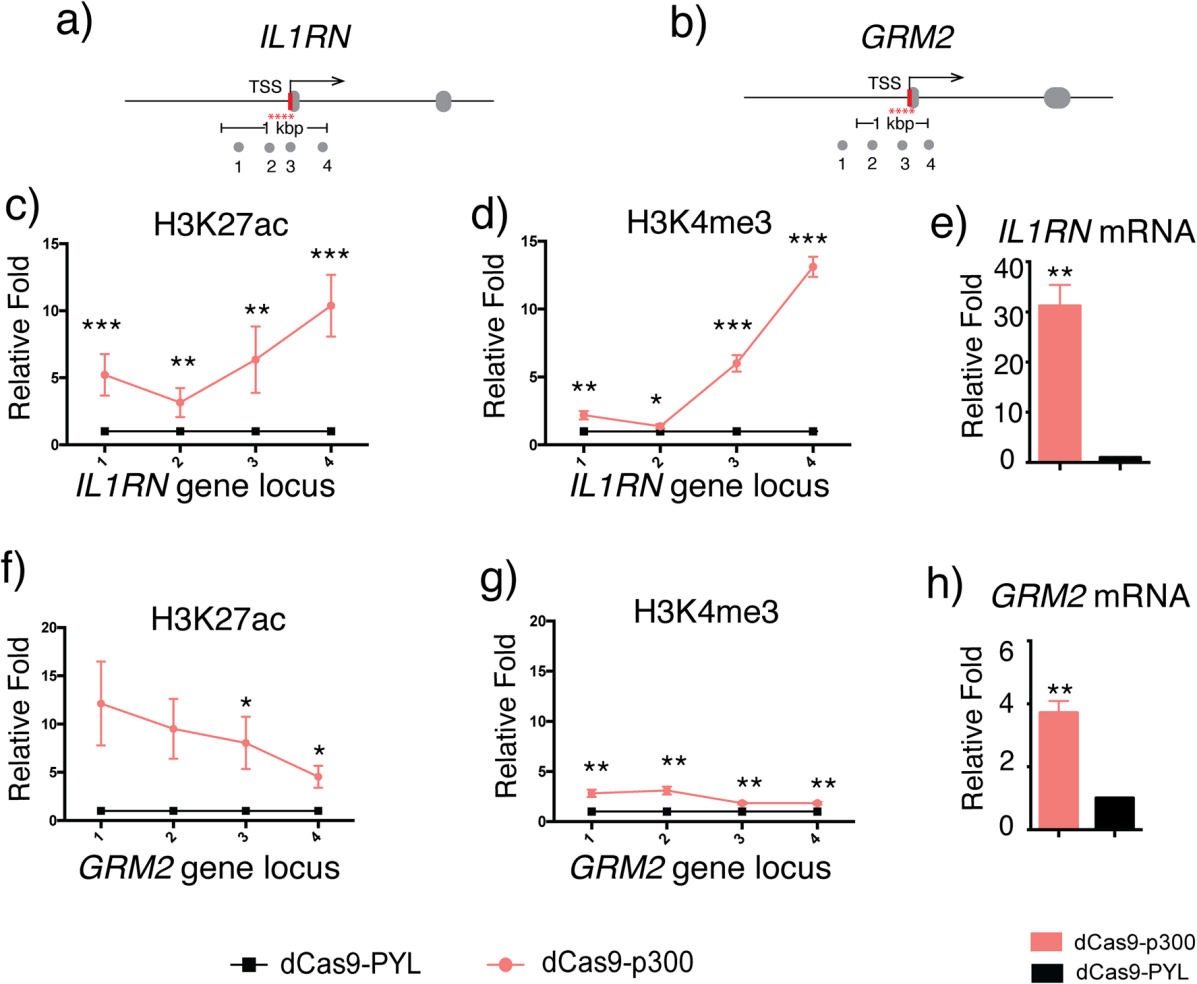
Locus-specific writing of H3K27ac induced by dCas9-p300. (**a**) and (**b**) The sites targeted by sgRNAs (red stars) and locations probed by qPCR amplicon (grey solid circles) at the *IL1RN* and *GRM2* loci. Enrichment of H3K27ac written induced by dCas9-p300 targeting at (**c**) *IL1RN* and (**f**) *GRM2* loci. Enrichment of H3K4me3 at (**d**) *IL1RN* and (**g)** *GRM2* loci upon targeted dCas9-p300 editing of H3K27ac. Changes in mRNA expression levels of (**e**) *IL1RN* and (**h**) *GRM2* upon induced H3K27ac writing. Fold changes of H3K27ac or H3K4me3 enrichment and mRNA level changes were calculated by normalizing results to those from samples of dCas9-PYL. Error bars represent ± s.e.m. from biological replicates (n = 6 for **c** and **d**, n = 3 for **e**–**h**). The *p* value less than 0.05 was marked as *, less than 0.01 as ** and less than 0.001 as ***.

To investigate the crosstalk between H3K27ac and H3K4me3 and their roles in gene activation, we used CRISPR/dCas9-based methods to recruit corresponding histone PTM modifying enzyme domains to desired gene loci. To artificially install H3K27ac at the promoter region, we used a previously reported dCas9-p300 acetyltransferase fusion protein^33^. Another dCas9 fusion protein, dCas9-PYL^34^, which has been used in our previous studies and does not have any histone PTM editing activity or known interactions with other chromatin modifying proteins, was used as a negative control. We transfected HEK293T cells with the dCas9-p300 or dCas9-PYL plasmids along with reported sgRNAs (a combination of four sgRNAs for each gene target) to target the promoters of *IL1RN*^33,34^ (Fig. 2a) or *GRM2*^34^ gene locus (Fig. 2b), which has been studied previously and validated for H3K27ac editing by CRISPR/dCas9-based methods. 72 h after transfection, cells were harvested and analyzed by chromatin immunoprecipitation (ChIP) assays using anti-H3K27ac or anti-H3K4me3 antibodies followed by quantitative polymerase chain reaction (qPCR) assays using primers designed to determine the levels of enrichment at indicated genome loci surrounding the promoter region and TSS under each condition (Fig. 2a,b). Consistent with previous results, dCas9-p300 increased H3K27ac levels around targeted loci on both *IL1RN* (Fig. 2c)^33^ and *GRM2* (Fig. 2f). Importantly, we observed the enrichment of H3K4me3 at *IL1RN* (Fig. 2d) and *GRM2* (Fig. 2g) loci as a result of dCas9-p300 targeting, which suggested that H3K27ac writing can lead to subsequent H3K4me3 installation. We also observed that the expression of *IL1RN* and *GRM2* genes were activated upon H3K27ac writing as reported^33,34^, determined by the increased mRNA levels of both genes using qPCR assays (Fig. 2e,h). To confirm the observed enrichment of H3K27ac was not caused by the overexpression of the dCas9-P300 fusion protein, we transfected cells with dCas9-P300 combined with either non-targeting (NT) sgRNA or *IL1RN*-specific sgRNA. The condition of dCas9-PYL with IL1RN sgRNA was also included as a control. From the same assays described above, we observed that the dCas9-P300 with NT-sgRNA did not increase the H3K27ac (Fig. S2a) or the H3K4me3 (Fig. S2b) level at the *IL1RN* locus. In addition, the *IL1RN* mRNA level was not increased under the same condition (Fig. S2c). These results indicated that the H3K27ac writing and the mRNA level increase were resulted from the site-specific recruitment of dCas9-P300 instead of the non-specific overexpression of P300.

### H3K4me3 writing cannot induce H3K27ac installation or activate transcription

**Figure 3:**
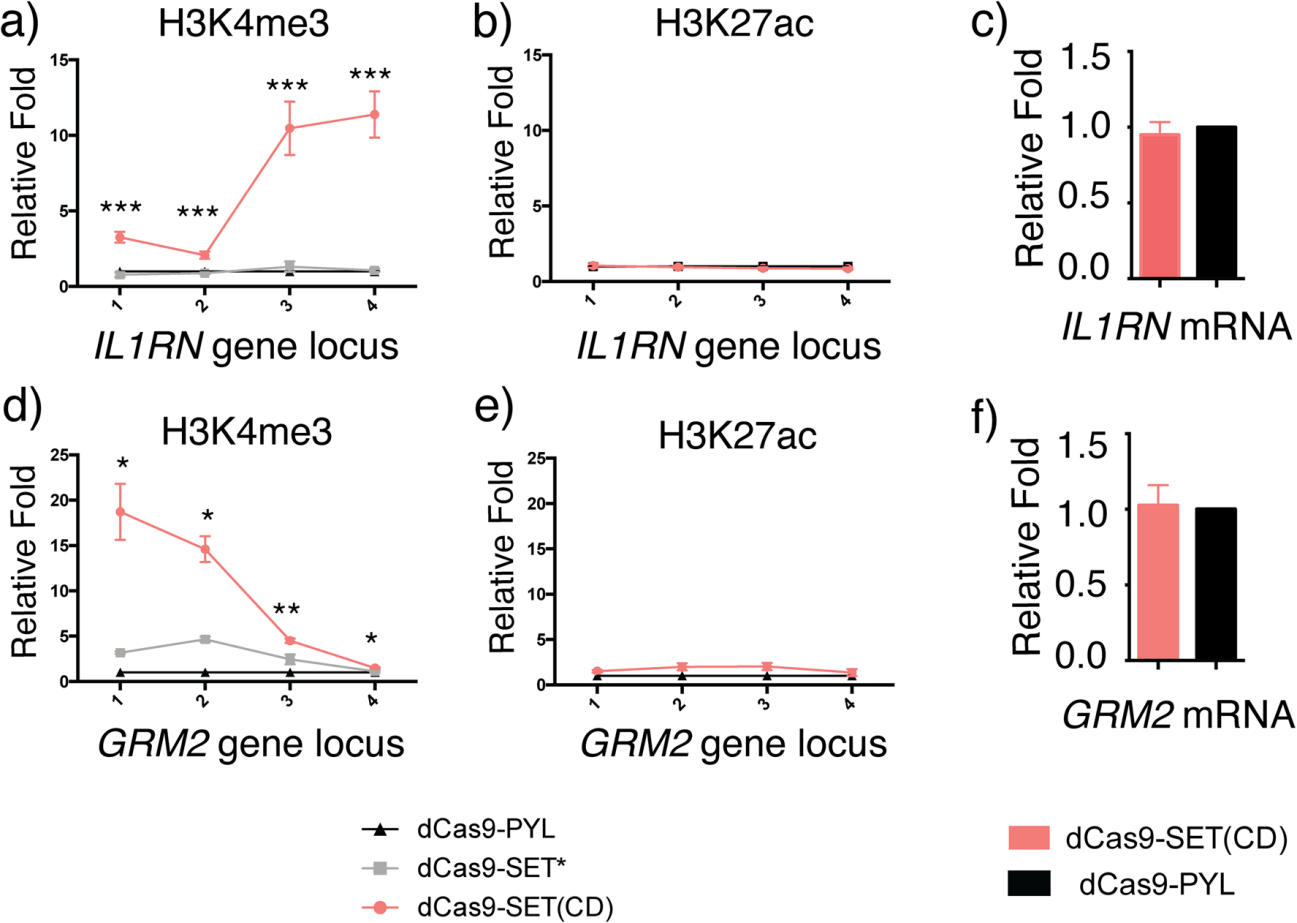
Locus-specific writing of H3K4me3 induced by dCas9-SET(CD). Enrichment of H3K4me3 at (**a**) *IL1RN* and (**d**) *GRM2* loci upon dCas9-SET(CD) (or dCas9-SET*) targeted H3K4me3 editing. Enrichment of H3K27ac at (**b**) *IL1RN* and (**e**) *GRM2* loci upon targeted dCas9-SET editing of H3K4me3. Changes in mRNA expression levels of (**c**) *IL1RN* and (**f**) *GRM2* upon induced H3K4me3 writing. Fold changes of H3K27ac and H3K4me3 enrichment as well as mRNA level changes were calculated by normalizing results to those from samples of dCas9-PYL. Error bars represent ± s.e.m. from biological replicates (n = 6 for **a**,**b**; n = 3 for **c**, f; n = 2 for **d**,**e**). The *p* value less than 0.05 was marked as *, less than 0.01 as ** and less than 0.001 as ***.

To investigate the crosstalk between H3K27ac and H3K4me3, and the effects of H3K4me3 on gene activity, we designed and cloned a fusion protein of dCas9 and the minimal n-SET catalytic domain of the H3K4me3-specific histone methyltransferase SET1A (termed as SET(CD)) for H3K4me3 writing. Previously reported dCas9-based H3K4me3 editors used the SET domain from PRDM9^35^, which included an SSX repression domain (SSXRD) and may cause transcription repression^40^. We decided to use the minimal SET domain (containing n-SET, SET and post-SET subdomains) without additional motifs to remove any functions that may interfere with H3K4me3 editing and complicate the resulting gene activity^37^. Meanwhile, a dCas9-fusion with catalytically inactivated SET(CD) mutant (S208I)^39^ (termed as dCas9-SET*) was also cloned to confirm the H3K4me3 writing was indeed resulted from the n-SET domain methyltransferase activity^37^. The expression of these dCas9 fusion proteins in cells were confirmed by western blotting (Fig. S1). To test H3K4me3 editing, HEK293T cells were transfected with plasmids encoding dCas9-SET(CD) or dCas9-SET* with the 4 sgRNAs^33,34^ targeting the *IL1RN* or *GRM2* promoter. After 72 h, cells were harvested and H3K4me3 and H3K27ac levels were determined by ChIP-qPCR assays using anti-H3K4me3 or anti-H3K27ac antibodies and primers probing the loci around the promoter and TSS of the *IL1RN* or *GRM2* genomic locus (Fig. 2a,b). We observed a significant enrichment of H3K4me3 at the probed genomic loci of both genes when cells were transfected with dCas9-SET(CD) as compared to the conditions of cells transfected with either dCas9-SET* or dCas9-PYL (Fig. 3a,d). These results suggested that the engineered dCas9-SET(CD) is fully functional for H3K4me3 writing and the methyltransferase activity is mainly responsible for the editing. Surprisingly, although H3K27ac writing was observed to promote H3K4me3 enrichment (Fig. 2d,g), we did not observe a significant increase of H3K27ac level at probed genomic loci of either *IL1RN* (Fig. 3b) or *GRM2* (Fig. 3e) upon H3K4me3 writing induced by dCas9-SET(CD). Furthermore, although the whole genome mapping had revealed that H3K4me3 is strongly correlated with active promoters and transcribed genes, other studies showed that H3K4me3 does not play an instructive role in gene activation^41–46^. Previous studies using CRISPR/dCas9 methods showed that inducing H3K4me3 writing was able to re-activate certain silence genes if the targeted genome loci were accessible^35^. However, in our dCas9-SET(CD) systems, even though we could successfully install H3K4me3, we did not observe the transcriptional activation of *IL1RN*, *GRM2* genes, based on the mRNA level changes of these genes determined using RT-qPCR (Fig. 3c,f).

To confirm the observed enrichment of H3K4me3 was not due to dCas9-SET(CD) overexpression, we transfected cells with dCas9-SET(CD) along with either non-targeting (NT) sgRNA or *IL1RN*-specific sgRNA. We observed that the dCas9- SET(CD) with NT-sgRNA did not lead to changes of the H3K4me3 (Fig. S3a) or H3K27ac (Fig. S3b) level at the *IL1RN* locus, nor the level of *IL1RN* mRNA (Fig. S3c). These results indicated that the H3K4me3 writing was resulted from the site-specific recruitment of dCas9-SET(CD) instead of the non-specific overexpression of SET(CD). In addition, to examine if the same relationship between H3K4me3 and H3K27ac can be observed in other gene locus, we performed the same editing experiments at the *MYOD1* locus^33,34^ (another confirmed locus for CRISPR-based targeting) using dCas9-SET(CD) and *MYOD1* sgRNAs^34^ (Fig. S4a). We observed that although H3K4me3 was enriched (Fig. S4b), the levels of H3K27ac (Fig. S4c) and *MYOD1* mRNA (Fig. S4d) were not changed similar to the results from the *IL1RN* and *GRM2* loci.

Overall, the observed results suggested that deposition of H3K27ac can induce the writing of H3K4me3, but not the other way around, and H3K27ac is sufficient to activate the expression of the tested genes but not H3K4me3. Collectively, it suggested that H3K27ac acts upstream to H3K4me3 and plays a more significant role in transcriptional activation.

### Inhibiting H3K27ac-BRD interactions by JQ1 impaired transcription

**Figure 4:**
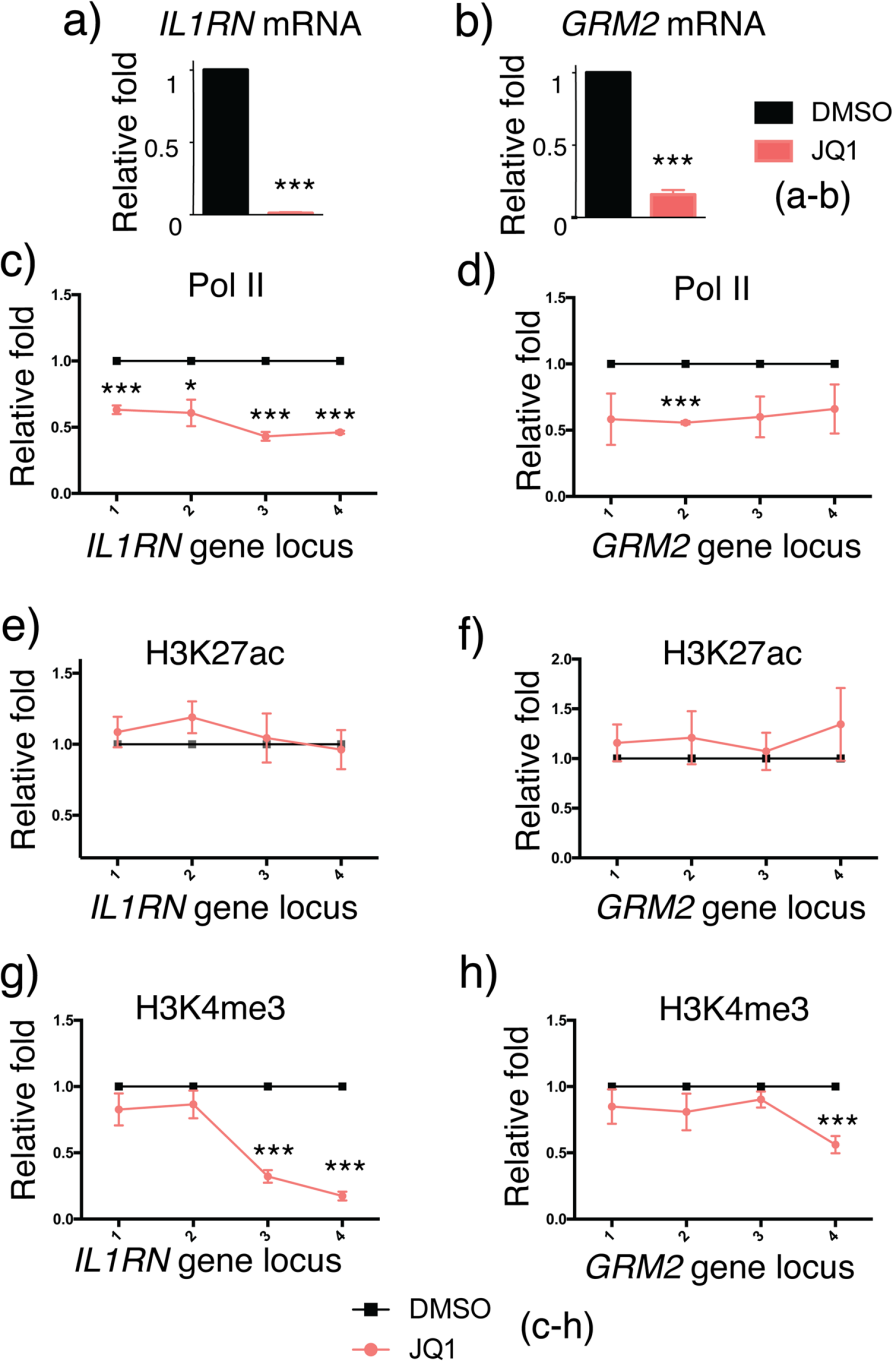
Effects of JQ1 treatment in dCas9-p300-induced H3K27ac writing and gene activation. The mRNA level changes of (**a**) *IL1RN* and (**b**) *GRM2* under dCas9-p300 targeting with or without JQ1 treatment. The enrichment fold changes of RNA polymerase II at (**c**) *IL1RN* and (**d**) *GRM2* loci upon JQ1 treatment. The enrichment fold changes of H3K27ac at (**e**) *IL1RN* and (**f**) *GRM2* loci upon JQ1 treatment. The enrichment fold changes of H3K4me3 at (**g**) *IL1RN* and (**h**) *GRM2* loci upon JQ1 treatment. Fold changes of Pol II, H3K27ac and H3K4me3 enrichment as well as the mRNA level changes were calculated by normalizing results to those from samples of DMSO treatment. Error bars represent ± s.e.m. from biological replicates (n = 3 for **a–d**; n = 4 for **e**,**f**; n = 5 for **g**,**h**). The *p* value less than 0.05 was marked as *, less than 0.01 as ** and less than 0.001 as ***.

We next investigated the mechanism of crosstalk between H3K27ac and H3K4me3 during CRISPR/dCas9-based epigenetic editing and transcriptional activation processes. The bromodomains and extra-terminal domain (BET) family proteins, including BRD2 and BRD4, are readers that recognize histone acetylation and recruit corresponding cellular machineries to initiate downstream molecular events, including transcription^47–49^. Previous studies showed that the recognition of H3K27ac at enhancers by BET proteins promoted the binding of super elongation complex to chromatin and lead to transcriptional activation^10^. JQ1 is a potent and selective inhibitor of BET bromodomains. It blocks the binding of readers to acetylated histones and suppress subsequent gene expression^50,51^. To confirm dCas9-p300-installed H3K27ac was indeed responsible for the observed gene activation through BRD binding, we tested if blocking BRD-H3K27ac recognition abolishes downstream H3K4me3 writing and gene activation. HEK293T cells transfected with dCas9-p300 and sgRNAs targeting *IL1RN* or *GRM2* locus for 24 h were treated with 2 $\mu$M JQ1^52^ (or DMSO as a control) for another 24 h. Cells were then harvested and subjected to subsequent qPCR assays to determine the changes in *IL1RN* and *GRM2* gene activities, and ChIP-qPCR assays to quantify the level changes of H3K27ac and H3K4me3. We observed that the JQ1 treatment abolished the dCas9-p300/H3K27ac-induced expression of *IL1RN* and *GRM2* when compared to the DMSO treatment conditions (Fig. 4a,b). RNA polymerase II (Pol II) is known to be enriched at the TSS of active genes. Using anti-Pol II antibody and ChIP-qPCR assays, we observed decreased Pol II levels at the *IL1RN* and *GRM2* loci upon JQ1 treatment under the conditions of dCas9-p300 targeting (Fig. 4c,d). These results indicated the pivotal role of H3K27ac in gene activation in the dCas-p300 editing system. In addition, although we did not observe H3K27ac level changes upon JQ1 treatment at either *IL1RN* or *GRM2* locus (Fig. 4e,f), we did observe decreases of H3K4me3 levels upon JQ1 treatment at both loci when compared to the DMSO treatment condition (Fig. 4g,h). These results again confirmed that H3K4me3 writing occurred downstream to H3K27ac writing.

### BRD2 mediated downstream effects of the artificially installed H3K27ac

BRD2 and BRD4 are two of the histone acetylation readers in mammalian BET family proteins and have been reported to have distinct functions^53^. It has been reported that BRD4 not only recognizes histone acetylation, but also enhances p300 histone acetyltransferase (HAT) activity through protein–protein interactions outside p300 catalytic HAT domain^52^. It was shown that the inhibition of BRD4 by JQ1 lead to the reduction of p300 activity and the global downregulation of H3K27ac^52^. However, we did not observe level changes in dCas9-p300-induced H3K27ac upon JQ1 treatment at the targeted *IL1RN* or *GRM2* loci, which may due to the fact that only the p300 HAT domain was used in the dCas9-p300 fusion protein. Alternatively, it has also been shown that BRD2 does not share the same property as BRD4 in stimulating p300 HAT activity^52^. It is possible that BRD2, instead of BRD4, functions as the reader for the artificially installed H3K27ac induced by the dCas9-p300 at the tested genomic loci.

**Figure 5:**
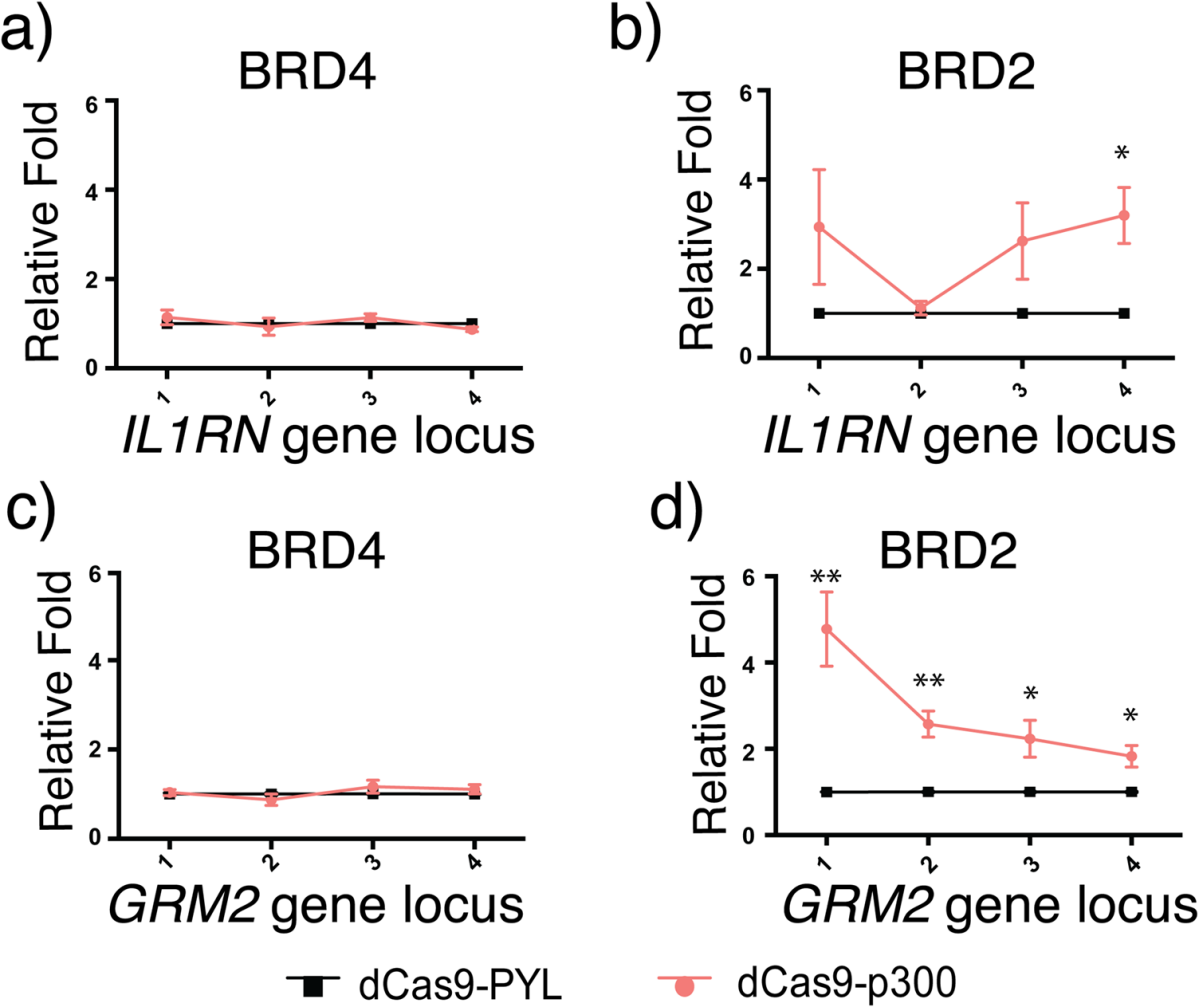
Recruitment of BRD proteins to dCas9-p300 targeted genome loci. Enrichment of BRD4 at (**a**) *IL1RN* and (**c**) *GRM2* loci upon dCas9-p300 targeting. Enrichment of BRD2 at (**b**) *IL1RN* and (**d**) *GRM2* loci upon dCas9-p300 targeting. Fold changes of BRD2 and BRD4 enrichment were calculated by normalizing results to those from samples of dCas9-PYL. Error bars represent ± s.e.m. from biological replicates (n = 3; **a**,**b**; n = 5 for **c**; n = 4 for **d**). The *p* value less than 0.05 was marked as *, less than 0.01 as ** and less than 0.001 as ***.

To determine if BRD2 or BRD4 was the reader for H3K27ac installed by the dCas9-p300 and mediated the downstream gene activation, we examined if the levels of BRD2 or BRD4 changed upon dCas9-p300-induced H3K27ac writing at the *IL1RN* and *GRM2* loci. HEK293T cells were transfected with plasmids encoding dCas9-p300 or dCas9-PYL with sgRNAs for *IL1RN* or *GRM2* locus and then harvested 48 h after transfection. The samples were subjected to ChIP-qPCR assays against BRD4 and BRD2 to evaluate their level changes at the designated loci (Fig. 2a,b). While we did not observe level changes in BRD4 at the probed genomic loci of *IL1RN* and *GRM2* when comparing dCas9-p300 versus dCas9-PYL conditions (Fig. 5a,c), BRD2 levels were significantly enriched when targeting dCas9-p300 to both loci over the dCas9-PYL control group (Fig. 5b,d). These results indicating BRD2, not BRD4 was recruited to these loci after H3K27ac installation and acted as the reader of H3K27ac in these dCas9-p300-induced processes.

**Figure 6:**
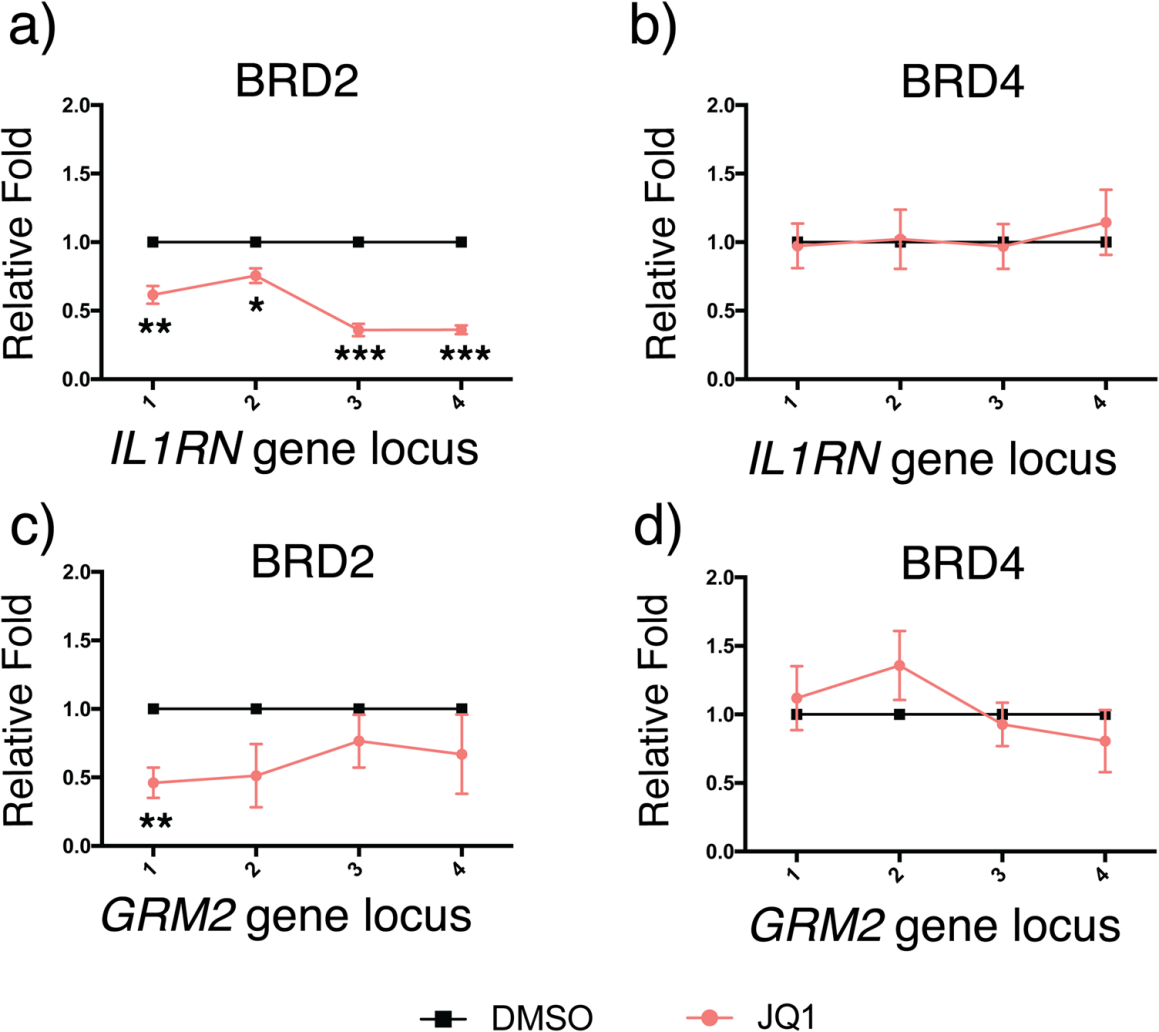
The effects of JQ1 on BRD recruitment upon dCas9-p300 targeting. Enrichment of BRD2 at (**a**) *IL1RN* and (**c**) *GRM2* loci upon JQ1 treatment under dCas9-p300 targeting. Enrichment of BRD4 at (**b**) *IL1RN* and (**d**) *GRM2* loci upon JQ1 treatment under dCas9-p300 targeting. Fold changes of BRD2 and BRD4 enrichment were calculated by normalizing results to those from DMSO-treated samples. Error bars represent ± s.e.m. from biological replicates (n = 3 for **a**; n = 5 for **b**,**d**; n = 4 for **c**). The *p* value less than 0.05 was marked as *, less than 0.01 as ** and less than 0.001 as ***.

To further confirm that BRD2 instead of BRD4 served as the reader of H3K27ac in these dCas9-p300-induced editing events, we treated dCas9-p300 transfected cells with JQ1 (or DMSO as a control) and performed ChIP-qPCR assays against BRD2 and BRD4 to examine the disruption on their recruitment to the *IL1RN* and *GRM2* by JQ1. We observed that the levels of BRD2 (Fig. 6a,c), but not BRD4 (Fig. 6b,d), were decreased at both probed gene loci. Based on these studies, it suggested that BRD2, instead of BRD4, was the H3K27ac reader that mediated H3K27ac-initiated downstream effects including H3K4me3 enrichment and the transcription of the *IL1RN* and *GRM2* genes.

## Discussion

In this study, we investigated the potential crosstalk between two active histone PTMs, H3K27ac and H3K4me3, and their roles in transcriptional regulation using CRISPR/dCas9-based epigenome editing approaches. We observed that H3K27ac writing at promoters through the dCas9-p300 editing system induced H3K4me3 installation around TSS and resulted in active transcription, whereas H3K4me3 artificially installed by dCas9-SET(CD) was unable to activate targeted genes or induce H3K27ac enrichment. BRD2, but not BRD4, was shown to be recruited after H3K27ac writing. The inhibition of BRD2 by JQ1 under the dCas9-p300-induced H3K27ac writing condition coincided with the abolished H3K4me3 installation and the impaired expression of dCas9-p300 targeted genes. Our results suggested that BRD2 translated the acetylation of H3K27 into transcriptional activation and potentially mediates the crosstalk between H3K27ac and H3K4me3 in the CRSIPR/dCas9-based epigenome editing systems^41,44–46,54,55^. However, we cannot rule out the possibility that H3K4me3 is a by-product of gene expression in our studies. Given the dynamic nature of epigenetic regulation in gene expression^4,56^, uncovering the relationships between different epigenetic marks and mechanisms requires new tools capable of resolving the temporal order and kinetics of molecular events occurred in these processes^10,31,57^. Inducible epigenome editing methods allowing precise temporal control have shown great potential in dissecting complex epigenetic mechanisms^31,34,58,59^. We anticipate that applying these temporally controlled technologies with genome targeting methods such as CRISPR methods can offer unparalleled insights in epigenetic functions and interplays of histone PTMs^34,58^.

## Methods

### Cell culture and transfection

HEK293T cells were maintained in Dulbecco’s modified Eagle’s medium (DMEM high glucose, Gibco 11,965,118) supplemented with 10% fetal bovine serum (Atlanta Biologicals S11550) in humid atmosphere containing 5% carbon dioxide. Cells were cultured at 37 °C. Cells were platted into 10 cm dishes or 24-well plates one day prior to transfection to allow the confluency of 70%-80% for subsequent transit transfection. For transfection of the cells in a 10 cm dish, 8 $\mu$g of dCas9 fusion plasmid DNA, 1 $\mu$g of each sgRNA and 36 $\mu$l PEI solution (1 mg/ml) were dissolved into 1 ml Opti-MEM (Gibco). For transfection of the cells in each well of a 24-well plate, 200–400 ng of dCas9 fusion plasmid DNA, 50 ng of each sgRNA and 1.2–1.8 $\mu$l PEI solution (1 mg/ml) were dissolved into 50 $\mu$l Opti-MEM (Gibco 51,985,091). After 15-mintue incubation at room temperature, the transfection reagents were gently added to the cell culture medium and were changed by fresh cell culture medium 4–6 h after transfection.

### Cloning of DNA constructs

The DNA fragment of the minimal SET catalytic domain of methyltransferase SET1A^60^ (amino acid: 1414–1707) was obtained by polymerase chain reactions (PCRs) with CloneAmp HiFi PCR Premix (Takara 639,298). The point mutation (S208I) of SET catalytic domain of SET1A was introduced through overlapping PCR approach.

SET(CD) F: 5’-GGCGCGCCGCCTACGAGCCACGCAGTGAG

SET(CD) R: 5’- GTTTAGGGAGCCCCGGCAGCT

SET*(S208I) F: 5’-TACGTGCAGGAGGGCATTGGCATTAGCTACCTGTTCCGGGTGGAC

SET*(S208I) R: 5’-GTCCACCCGGAACAGGTAGCTAATGCCAATGCCCTCCTGCACGTA

#### Chromatin immunoprecipitation (ChIP) assay

ChIP assays were performed with the guidance of SimpleChIP Plus Sonication ChIP protocol from Cell Signaling Technology (CST). To crosslink proteins to genomic DNA, the final concentration of 1% formaldehyde was gently added to the cell culture dish and incubated for 10 min at room temperature. Then 1 ml 1 M glycine was added into each 10 cm dish containing 10 ml cell culture medium and incubated for 5 min to quench the crosslink. The cell culture medium was gently removed and the fixed cells were washed twice with 10 ml cold PBS with 1X protease inhibitor cocktail (PIC, Thermo 78,430). Cells were scraped followed by centrifugation at 1000 g for 5 min at 4 °C to collect cell pellets. Chromatins were obtained with cell lysis buffer (CST 96,529) and nuclear lysis buffer (CST 28,778) according to the protocol. Chromatin was sonicated at 4 °C by Bioruptor Pico (Diagenode) with the program of 10 cycles of 30-s on and 30-s off. An IP reaction was set up with 410 $\mu$l 1X ChIP buffer (CST 7008), 90 l$\mu$ fragmented chromatins, corresponding ChIP grade antibody and 5 $\mu$l 1X PIC, and 2% of the reaction mixture was saved as input. The IP reaction was incubated overnight at 4 °C with rotation. Then 30 $\mu$l Protein G Magnetic Beads (CST 9006) were added into the IP sample for a subsequent 2-h at 4 °C. Beads isolated from each ChIP reaction were washed with 1 ml low salt buffer (1X ChIP buffer) for three times and one time with 1 ml high salt buffer (1X ChIP buffer with 70 l$\mu$ 5 M NaCl). Bound chromatin fragments were then eluted from the beads with 150 $\mu$l ChIP Elution buffer (CST 7009) and were subjected to further elution from antibody with 2 $\mu$l proteinase K proteinase K (Thermo 25,530,049) and 6 $\mu$l 5 M NaCl addition. Then the sample mixture was incubated at 65 °C for at least 2 h. The ChIP DNA is finally purified by DNA purification kit (CST 14,209) for subsequent qPCR analysis.

### Total RNA isolation and purification

Total RNA was isolated from harvested cells by Trizol reagent (Thermo 15,596,018) along with chloroform, isopropanol and 75% ethanol treatment according to the manufacture’s standard protocol. To extract RNA from cells seeded in 10-cm dish, cell culture medium was firstly removed and 2 ml Trizol reagent was added directly into the cell culture dish followed by pipetting the lysate several times. Cells with Trizol were collected into centrifuge tubes and were incubated at 4 °C for 3–5 min to allow complete dissociation of proteins and nucleic acids. 0.2 mL chloroform per 1 ml Trizol reagent was added to each tube. With the cap of microcentrifuge tube secured, the tube was shaken vigorously for 2 min and incubated at 4 °C for 10–15 min. Samples were centrifuged for 15 min at 4 °C and the aqueous phase containing RNA was transferred into a new tube. Next, 0.5 mL isopropanol per 1 mL Trizol reagent was added into the new tube with aqueous phase to precipitate RNA. The tube was incubated at 4 °C for 10–30 min followed by centrifugation at 13,500 rpm at 4 °C. Supernatant was discarded carefully and the gel-like crude RNA precipitate was saved and washed with 1 mL 75% ethanol per 1 ml Trizol reagent used. The RNA in ethanol was pelleted with centrifugation and air dried. The dried RNA was dissolved in 50–100 $\mu$l nuclease-free water and was subjected to subsequent reverse transcription experiments or saved in − 80 °C.

### Reverse transcription and qPCR assay

Reverse transcription was performed with 50–200 ng total RNA as template using iScript super mix (Bio-Rad 1,708,891). A 20 $\mu$l reaction mixture was set up with 4 $\mu$l 5 × reaction mix, 1 $\mu$l reverse transcriptase, nuclease-free water and 50–200 ng total RNA. The reverse transcription reaction was incubated in a thermal cycler (Bio-Rad) using the following protocol: priming for 5 min at 25 °C, reverse transcription for 25 min at 46 °C, inactivation for 1 min at 95 °C. The completed reaction containing cDNA was subjected to subsequent qPCR analysis or could be stored at − 20 °C.

The 0.5 $\mu$l DNA solution isolated from ChIP assay or 50–200 ng cDNA was applied as template and Ssoadvanced Universal SYBR green mix (Bio-Rad 1,725,274) was employed for subsequent qPCR reactions according to the users’ manual. A 10 $\mu$l reaction mixture was set up with 5 $\mu$l 2 × SYBR green mix, 1 $\mu$l of forward and reverse primer, nuclease-free water and DNA template. Multiplexed qPCR reactions were performed under the 384-well format in a 7900HT Fast Real-Time PCR System (Applied Biosystems), with all reactions being assayed at least triplicate for gene expression detection or at least duplicate for ChIP-qPCR analysis. Corresponding raw data were collected and processed with the complimentary SDS 2.4 software under default settings.

Further analysis of the Ct values was calculated as describe in our previous publication with minor modifications. For gene expression analysis, *GAPDH* was used as housekeeping gene internal control and was in parallel detected with a SYBR green mix (Bio-Rad 1,725,274) and in-house designed primers. In ChIP-qPCR analysis, 2% input was applied for further normalization and was in parallel assayed by a SYBR green mix (Bio-Rad 1,725,274) as suggested by the CST SimpleChIP Plus Sonication protocol.

The thermal profiles of qPCR reactions are listed below:

*Step 1* Polymerase activation and DNA denaturation for 30 s (cDNA) at 95 °C or for 3 min (genomic DNA/ChIP DNA).

*Step 2* Denaturation at 95 °C or 98 °C for 15 s.

*Step 3* Annealing/ extension and plate read at 60 °C for 1 min.

Go to step 2 and amplification for 40 cycles.

*Step 4* Dissociation at 95 °C for 15 s, then 60 °C for 1 min and 95 °C for 15 s.

### Statistical analysis

All of the above statistical analyses were performed by GRAPHPAD Prims 6 with default settings. In brief, statistical significance of differences between experimental and control groups in ChIP-qPCR assays and gene expression qPCR assays was evaluated by multiple *t*-test. In this manuscript, a *p* value less than 0.05 was marked as *, less than 0.01 as ** and less than 0.001 as ***. Detailed *p* values were listed in the supplementary information.

## Supplementary Information

Supplementary Information.
