# Supplementary material for: Investigating crosstalk between H3K27 acetylation and H3K4 trimethylation in CRISPR/dCas-based epigenome editing and gene activation
Source: Sci Rep. 2021 Aug 5;11:15912. doi: 10.1038/s41598-021-95398-5 (PMC8342468; doi:10.1038/s41598-021-95398-5)
Supplement: Supplementary file 1 — Supplementary Information. [file 41598_2021_95398_MOESM1_ESM.pdf]

Investigating Crosstalk Between H3K27 Acetylation and H3K4 Trimethylation in CRISPR/dCas-based Epigenome Editing and Gene Activation

Weiye Zhao‡, Ying Xu‡, Yufan Wang, Dan Gao, Jasmine King, Yajie Xu and Fu-Sen Liang*

Department of Chemistry, Case Western Reserve University, 2080 Adelbert Road, Cleveland, OH 44106, United States

* Corresponding author

Correspondence: fxl240@case.edu

‡ These authors contributed equally to this work

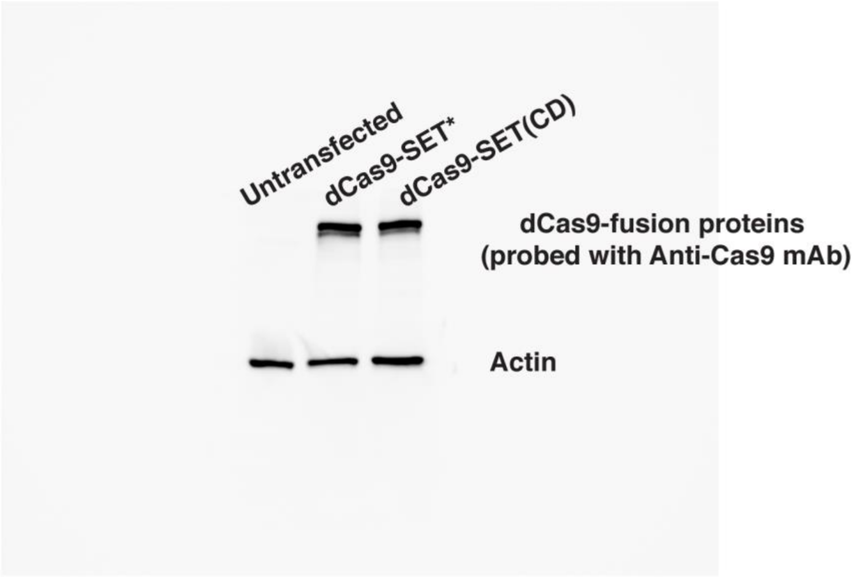

Supplementary Figure 1. Expression of dCas9-SET(CD) and dCas9-SET* fusions in HEK293T cells. Western blotting of the two dCas9 fusion proteins with Actin as the loading control (See **Supplementary Table 2** for antibodies used for the experiments).

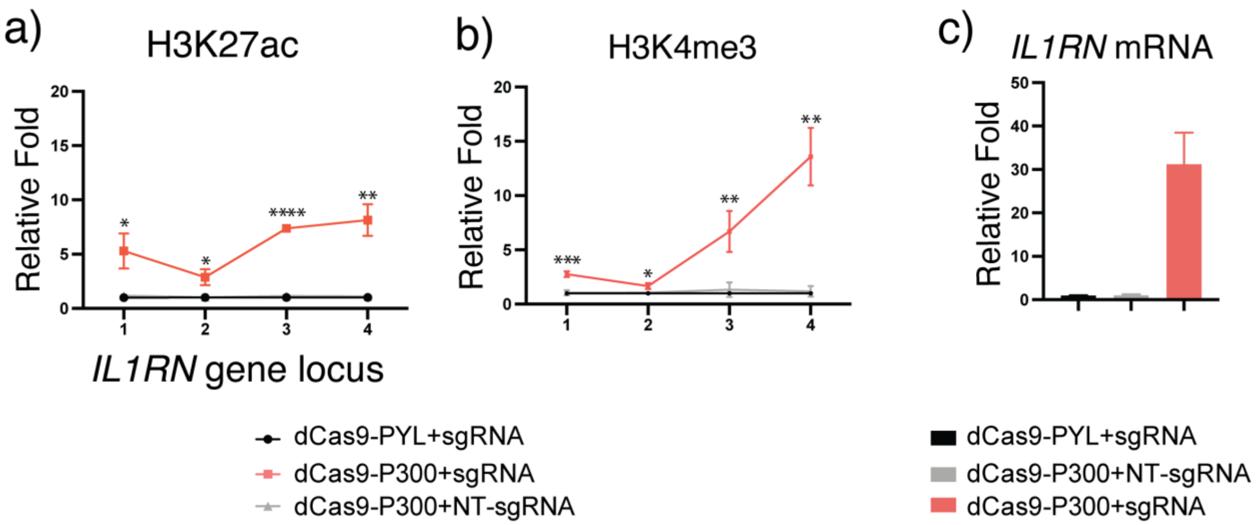

Supplementary Figure 2. Locus-specific writing of H3K27ac on IL1RN induced by dCas9-p300. a) Enrichment of H3K27ac on IL1RN loci induced by dCas9-p300 with NT-sgRNA or sgRNAs. b) Enrichment of H3K4me3 on IL1RN loci induced by dCas9-p300 with sgRNAs or NT-sgRNA. c) IL1RN mRNA level of cells transfected with dCas9-p300 with sgRNAs or NT-sgRNA. Fold changes of H3K27ac or H3K4me3 enrichment and mRNA level changes were calculated by normalizing results to those from samples of cells transfected with dCas9-PYL and sgRNAs.

Error bars represent ± s.e.m. from biological replicates (n = 3). The p value less than 0.05 was marked as *, less than 0.01 as ** and less than 0.001 as ***.

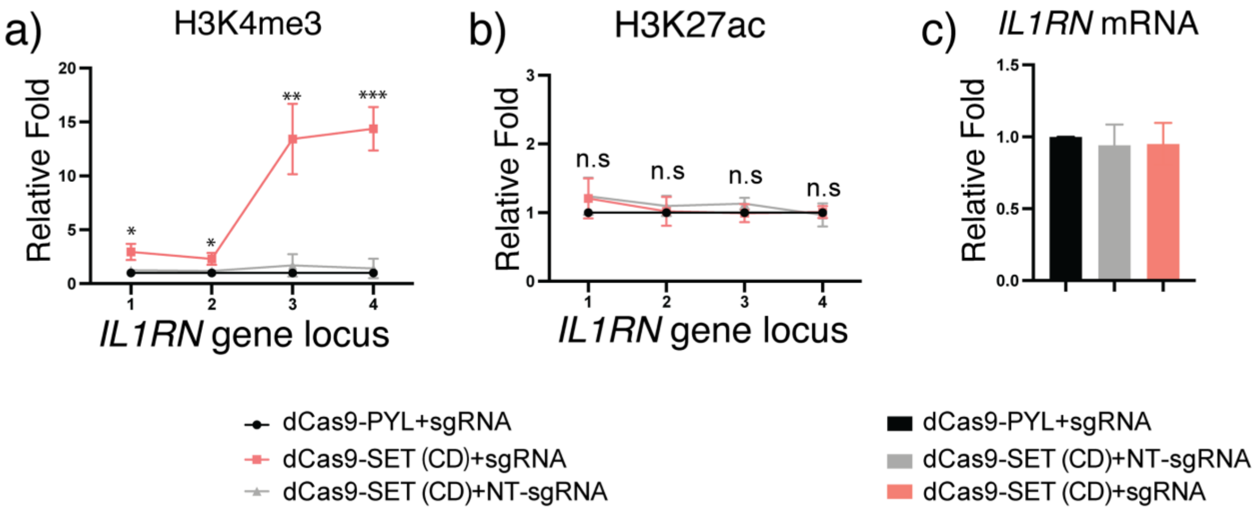

Supplementary Figure 3. Locus-specific writing of H3K4me3 on IL1RN induced by dCas9-SET(CD). a) Enrichment of H3K4me3 on IL1RN loci induced by dCas9- SET(CD) with NT-sgRNA or sgRNAs. b) Enrichment of H3K27ac on IL1RN loci induced by dCas9- SET(CD) with sgRNAs or NT-sgRNA. c) IL1RN mRNA level of cells transfected with dCas9- SET(CD) with sgRNAs or NT-sgRNA. Fold changes of H3K27ac or H3K4me3 enrichment and mRNA level changes were calculated by normalizing results to cells transfected with dCas9-PYL and

sgRNAs. Error bars represent ± s.e.m. from biological replicates (n = 3). The p value less than 0.05 was marked as *, less than 0.01 as ** and less than 0.001 as ***.

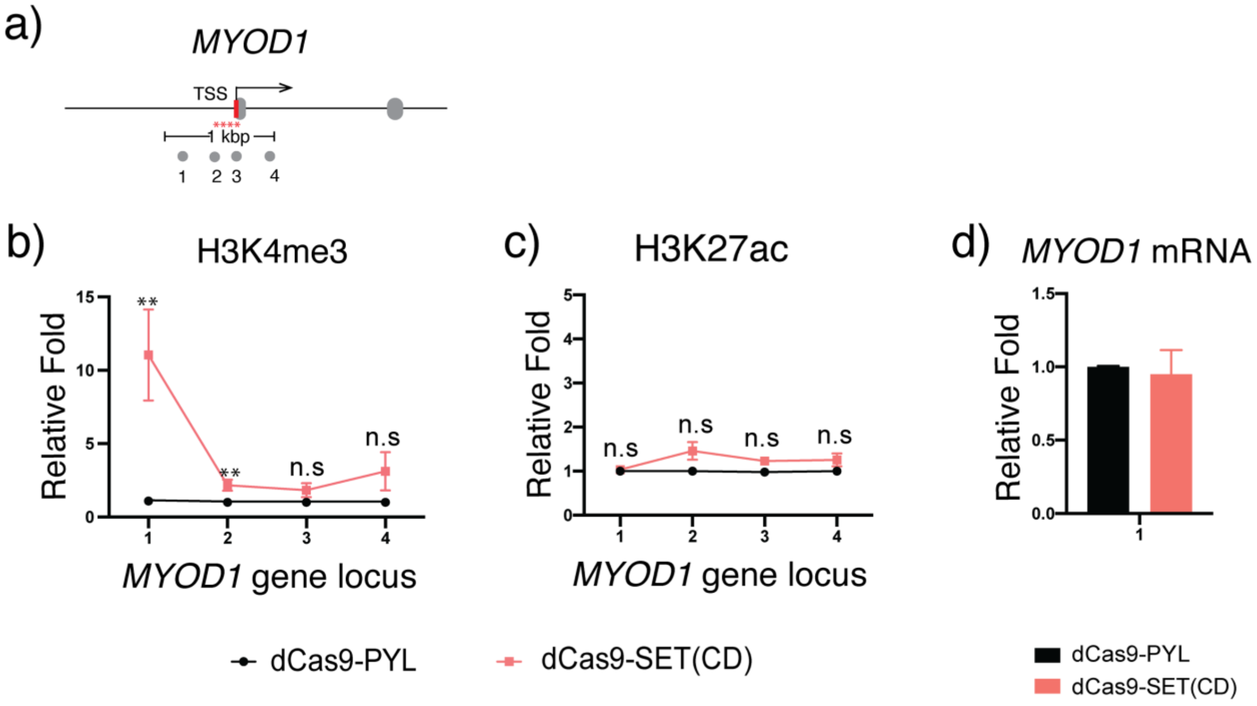

Supplementary Figure 4. Locus-specific writing of H3K27ac on MYOD1 induced by dCas9-SET(CD). a) Enrichment of H3K27ac on MYOD1 loci induced by targeted dCas9- SET(CD) editing. b) Enrichment of H3K4me3 on MYOD1 loci induced by dCas9- SET(CD) targeted

dCas9-SET(CD). c) MYOD1 mRNA level of cells transfected with targeted dCas9-SET(CD) editing. Fold changes of H3K27ac or H3K4me3 enrichment and mRNA level changes were calculated by normalizing results to cells transfected with dCas9-PYL and sgRNAs. Error bars represent ± s.e.m. from biological replicates (n = 3). The p value less than 0.05 was marked as *, less than 0.01 as ** and less than 0.001 as ***.

Supplementary methods

Western blotting

Western blotting was performed as descried in the Bio-Rad General Protocol for Western Blotting with minor modifications. Briefly, cells were lysed on ice with 1X RIPA buffer (Thermo 89900) supplemented with 1X protease inhibitor cocktail (Thermo 78430). Cell lysate was briefly sonicated for three times and was agitated for 30 minutes at 4 °C, followed by centrifugation at 12,000 rpm for 20 minutes at 4 °C. The supernatant of cell lysate was saved and was subjected to determine the protein concentration by colorimetric method. 20 μg protein was used for protein gel electrophoresis by following standard protocol. Proteins were transferred from gel to PVDF membrane previously activated by pure methanol at the constant current of 150 mA for 90 minutes. After blocking by TBST buffer containing 10% fat-free milk for one hour, the PVDF membranes with transferred proteins were incubated in the TBST buffers containing 10% fat-free milk with corresponding primary antibodies for two hours at room temperature or overnight at 4 °C. After being washed in fresh TBST buffer for three times,

membranes were incubated with corresponding HRP-conjugated secondary antibodies for one hour at room temperature. Membranes were washed in fresh TBST buffer for three times and protein blots were imaged with Western ECL Substrate (Bio-Rad 1705060) and Bio Rad ChemiDoc MP Imaging System.

DNA oligos and primers

Primers for qPCR assays in this chapter are listed below.[1]

GAPDH F: 5'- GGTGGTCTCCTCTGACTTCAACA (mRNA qPCR)

GAPDH R: 5'- GTTGCTGTAGCCAAATTCGTTGT (mRNA qPCR)

GRM2 F: 5'- CTTTGTGCTCAACGTCAAGTTT (mRNA qPCR)

GRM2 R: 5'-TTGTAGCGGCCAATACCATC (mRNA qPCR)

IL1RN F: 5'- GACCTTCTATCTGAGGAACAACC (mRNA qPCR)

IL1RN R: 5'- CACAGGACAGGCACATCTT (mRNA qPCR)

GRM2 ChIP 1F: 5'-AGTGTCGGGAATGTGTGTAG

GRM2 ChIP 1R: 5'-GGGTAGTGGATAAGCTGTAGTG

GRM2 ChIP 2F: 5'-CCCATTCTCCTCTGACCTCT

GRM2 ChIP 2R: 5'-CATGTGTCCTCTGCCACTTT

GRM2 ChIP 3F: 5'-GAGTGGGCCACGAAGCA

GRM2 ChIP 3R: 5'-TCCGCCTCCTCTGGACA

GRM2 ChIP 4F: 5'-GGGAAGCGGGAAGACAG

GRM2 ChIP 4R: 5'-CAGACAGAAAGAAGGACCAGAG

IL1RN ChIP 1F: 5'- GTTAGAGCGTTGGGGACCTT

IL1RN ChIP 1R: 5'- CACATGCAGAGAACTGAGCTG

IL1RN ChIP 2F: 5'- TTCTCTGCATGTGACCTCCC

IL1RN ChIP 2R: 5'- ACACACTCACAGAGGGTTGG

IL1RN ChIP 3F: 5'- GCTGGGCTCCTCCTTGTACT

IL1RN ChIP 3R: 5'- GCTGCTGCCCATAAAGTAGC

IL1RN ChIP 4F: 5'- CAGGTGAACAGAGAGGTGTAAC

IL1RN ChIP 4R: 5'- GGCTATTTCACCAATTTCCCTATTC

MYOD1 CHIP 1F: 5'- TCGGAGACTTCAGGTGAGAT

MYOD1 CHIP 1R: 5'- GGTGCTGAGTCAAGGAAAGT

MYOD1 CHIP 2F: 5'- GGGACAGAGGAGTATTGAAAGTC

MYOD1 CHIP 2R: 5'- CCCTTTCCAAACCTCTCCAA

MYOD1 CHIP 3F: 5'- TCCTATTGGCCTCGGACT

MYOD1 CHIP 3R: 5'- GCGCCCTGGGCTATTTA

MYOD1 CHIP 4F: 5'- CCGCCTGAGCAAAGTAAATG

MYOD1 CHIP 4R: 5'- CGATATAGCGGATGGCGTT

Supplementary Table 1. sgRNA **target** sequences

Target Location	Protospacer Sequence (5'-3')
IL1RN sgRNA1	TGTACTCTCTGAGGTGCTC
IL1RN sgRNA2	ACGCAGATAAGAACCAGTT
IL1RN sgRNA3	CATCAAGTCAGCCATCAGC
IL1RN sgRNA3	GAGTCACCCTCCTGGAAAC
GRM2 sgRNA1	GGATAGGTAAAGGGGCGCGT
GRM2 sgRNA2	GAAGGTCACTGCGCCCCGAC
GRM2 sgRNA3	GCGCAGAGCGAGAGCGCTCG

GRM2 sgRNA4	GTCTGACTATGGGGCGGAGT
MYOD1 sgRNA1	CCTGGGCTCCGGGGCGTTT
MYOD1 sgRNA2	GGCCCCTGCGGCCACCCCG
MYOD1 sgRNA3	CTCCCTCCCTGCCCGGTAG
MYOD1 sgRNA4	AGGTTTGGAAAGGGCGTGC

Supplementary Table 2. Antibodies used in the manuscript

Target Protein	Application	Antibody	Catalog number	Vendor
H3K27ac	ChIP	Acetyl-Histone H3 (Lys27) (D5E4) XP Rabbit mAb	8173	CST
H3K4me3	ChIP	Tri-Methyl-Histone H3 (Lys4) (C42D8) Rabbit mAb	9751	CST
BRD4	ChIP	BRD4 (E2A7X) Rabbit mAb	3440	CST
BRD2	ChIP	Brd2 (D89B4) Rabbit mAb	5848	CST

RNA polymerase II	ChIP	Anti-RNA polymerase II CTD repeat YSPTSPS antibody	ab26721	Abcam
dCas9-fusions	Western blotting	Cas9 (S. pyogenes) (E7M1H) XP Rabbit mAb	19526	CST
Rabbit IgG (primary antibodies)	Western blotting	Anti-rabbit IgG, HRP-linked Antibody	7074	CST
Mouse IgG (primary antibodies)	Western blotting	Anti-mouse IgG, HRP-linked Antibody	7076	CST
Actin	Western blotting	Anti-Actin Antibody, clone C4	MAB1501	Millipore

Supplementary Table 3. P-values of multiple t-test statistical analysis.

Figure	Locus 1	Locus 2	Locus 3	Locus 4
Fig. 2c	0.000358342	0.00315147	0.00122975	9.300070e-005
Fig. 2d	0.00329701	0.0448215	8.559349e-006	1.533925e-008

Fig. 2f	0.0624506	0.0518708	0.0398115	0.0349431
Fig. 2g	0.00798849	0.00589646	0.00262888	0.00379617
Fig. 3a	9.021050e-005	0.000727447	0.000309756	4.782524e-005
Fig. 3b	0.702656	0.555236	0.0965662	0.0812065
Fig. 3d	0.0291481	0.0107982	0.00438154	0.0336188
Fig. 3e	0.0651301	0.12048	0.120816	0.454054
Fig. 4c	0.000334383	0.0172333	6.834936e-005	1.158602e-006
Fig. 4d	0.195193	0.000102569	0.13718	0.251354
Fig. 4e	0.453724	0.140265	0.807797	0.794666
Fig. 4f	0.427042	0.461455	0.714493	0.384061
Fig. 4g	0.191953	0.233093	8.406288e-007	7.279145e-009
Fig. 4h	0.278173	0.202313	0.149404	0.000148974
Fig. 5a	0.434591	0.750012	0.183235	0.0705202
Fig. 5b	0.206657	0.472941	0.130717	0.0249652
Fig. 5c	0.637228	0.35431	0.307055	0.315706
Fig. 5d	0.00461213	0.00198737	0.027619	0.0161649
Fig. 6a	0.0040776	0.0105566	0.000135884	3.819284e-005
Fig. 6b	0.888341	0.931784	0.870483	0.60722
Fig. 6c	0.00278734	0.0530454	0.269275	0.297452

Fig. 6d	0.622257	0.192909	0.659935	0.41804
Fig. 2e	0.00196258			
Fig. 2h	0.00186871			
Fig. 3c	0.590364			
Fig. 3f	0.845678			
Fig. 4a	6.277860e-009			
Fig. 4b	1.242010e-005			

1 Chen, T. *et al.* Chemically Controlled Epigenome Editing through an Inducible dCas9 System. *Journal of the American Chemical Society* **139**, 11337-11340, doi:10.1021/jacs.7b06555 (2017).
